# Accurate Vertical Ionization Energy of Water and Retrieval of True Ultraviolet Photoelectron Spectra of Aqueous Solutions

**DOI:** 10.1021/acs.jpclett.2c01768

**Published:** 2022-07-21

**Authors:** Michael S. Scholz, William G. Fortune, Omri Tau, Helen H. Fielding

**Affiliations:** Department of Chemistry, University College London, 20 Gordon Street, London WC1H 0AJ, United Kingdom

## Abstract

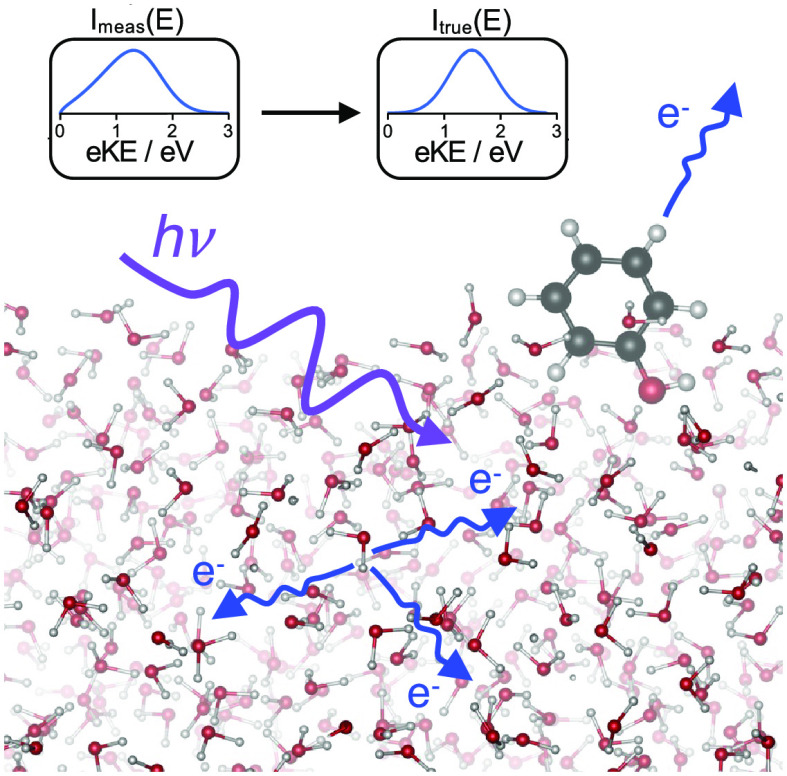

Ultraviolet (UV) photoelectron spectroscopy provides a direct way of measuring valence electronic structure; however, its application to aqueous solutions has been hampered by a lack of quantitative understanding of how inelastic scattering of low-energy (<5 eV) electrons in liquid water distorts the measured electron kinetic energy distributions. Here, we present an efficient and widely applicable method for retrieving true UV photoelectron spectra of aqueous solutions. Our method combines Monte Carlo simulations of electron scattering and spectral inversion, with molecular dynamics simulations of depth profiles of organic solutes in aqueous solution. Its application is demonstrated for both liquid water, and aqueous solutions of phenol and phenolate, which are ubiquitous biologically relevant structural motifs.

Aqueous solutions play a central role in chemistry; however, determining their electronic structure is still challenging. One of the most direct ways of determining electronic structure is to use photoelectron spectroscopy (PES) to measure electron binding energies (eBEs). For a long time, PES was restricted to the gas or solid phases, due to the requirement for high vacuum to minimize scattering of the emitted electrons.^1,2^ However, the introduction of liquid jets and their combination with intense X-ray sources at synchrotrons, in the late 1990s,^3^ expanded the scope of PES to include liquids. Liquid-jet PES (LJ-PES) is now an active research field involving a growing number of research groups around the world.^4−14^ Nonetheless, the requirement for relatively high concentrations of solute to obtain adequate signal-to-noise ratio after subtracting the photoelectron spectrum of water has excluded most X-ray LJ-PES studies of aqueous solutions of organic molecules because organic molecules tend to be only weakly soluble.^9^ A solution to this problem is to use resonance-enhanced PES with ultraviolet (UV) light pulses.^9,14−23^ Unfortunately, a major challenge that has been hampering the development of UV LJ-PES for aqueous solutions is a lack of consensus on the precise effect of inelastic scattering of low kinetic energy electrons in liquid water on peak shapes and positions.^24−30^

A schematic illustration of the inelastic scattering process following resonance-enhanced photoionization/detachment in aqueous solution is shown in Figure 1. The initial photoelectron kinetic energy (eKE) distribution in the conduction band, *I*_true_(*E*), is formed with a spatial distribution defined by the laser focusing conditions and the solute concentration depth profile. Subsequent transmission through the conduction band results in distortion of the initial eKE distribution due to inelastic scattering of the electrons from water molecules. Consequently, the peak of the measured distribution, *I*_meas_(*E*), is shifted to lower eKE, which introduces inaccuracies in eBEs extracted from such measurements. Transmission through the water/vacuum interface and the spectrometer result in further distortions; the electrons require a threshold eKE to escape the liquid and the transmission efficiency of the spectrometer drops for electrons with very low eKE (≲0.3 eV in our spectrometer).

**Figure 1:**
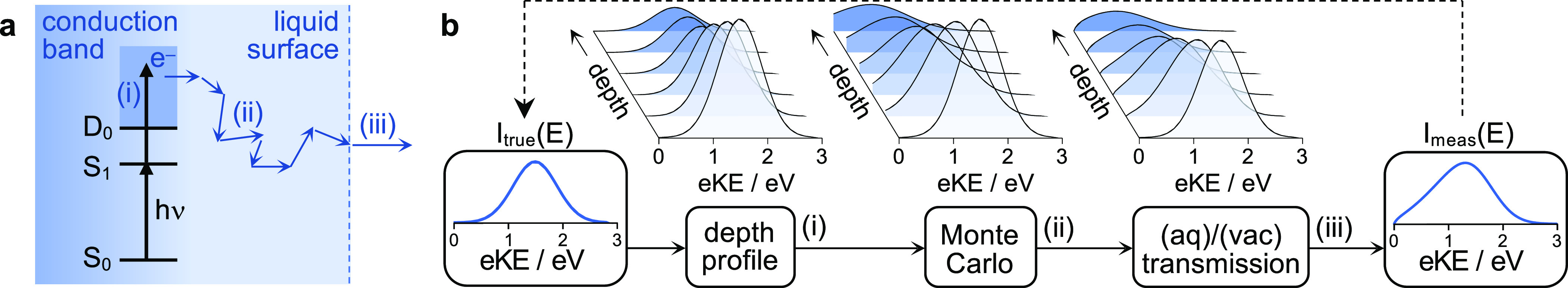
(a) Energy diagram of multiphoton photoionization/photodetachment and schematic illustration of subsequent electron transport in aqueous solution: (i) initial eKE distribution in the conduction band, *I*_true_(*E*); (ii) electron scattering; (iii) transport through the water–vacuum interface and spectrometer to generate the measured eKE distribution, *I*_meas_(*E*). (b) Flow diagram of key simulation steps (bottom) and cartoons (top) illustrating the *I*_true_(*E*) → *I*_meas_(*E*) transformation (solid arrows) and spectral retrieval (dashed arrow).

So far, two approaches have been employed to extract accurate eBEs of solvated electrons (*e*_aq_^–^) from UV LJ-PES measurements. (1) Monte Carlo simulations using electron scattering cross-sections determined from photoelectron spectroscopy measurements of liquid droplets^31^ have been employed to model a series of UV photoelectron spectra of *e*_aq_^–^.^25^ Although the simulated true eBE distribution had an unexplained shoulder on the high-eBE side, which has generated some discussion,^27−29,32^ it had the same peak maximum as EUV photoelectron spectra of *e*_aq_^–^ that were measured subsequently.^26^ A slight disadvantage of the method is that it employs an iterative procedure involving repeated Monte Carlo simulations in a grid search for fitting parameters and is therefore computationally intensive and requires parallel computing resources. (2) A spectral inversion process based on the assumption that extreme UV (EUV) LJ-PES measurements yield the true eKE distribution has been employed to retrieve true UV photoelectron spectra from measured UV photoelectron spectra.^26,33,34^ Although recent work has suggested that inelastic scattering and indirect autoionization processes distort the distributions obtained from EUV LJ-PES measurements using photon energies < 30 eV above the ionization threshold,^35^ the peak maximum was the same as that obtained using the Monte Carlo simulations.^25^ A slight disadvantage of this approach is that its wide applicability is limited by the extremely low signals obtained from EUV LJ-PES measurements of weakly soluble solutes and by experimental complexity. It is also worth noting that neither of these methods considered, explicitly, non-uniform solute concentration depth profiles, which are common for weakly soluble organic molecules that tend to have an enhanced surface concentration.^14,36,37^

Here, we present a computationally efficient and widely applicable method for retrieving true photoelectron spectra from UV LJ-PES measurements of aqueous solutions with arbitrary depth profiles. Inspired by the methods developed to extract accurate eBEs of solvated electrons,^25,26^ we combine spectral inversion with Monte Carlo simulations of electron scattering in water to determine *I*_true_(*E*) → *I*_meas_(*E*) linear transformations, together with molecular dynamics simulations of depth profiles of solutes in aqueous solution. We demonstrate the applicability of the approach by retrieving true photoelectron spectra of liquid water,^4,12,38−41^ and aqueous solutions of phenol and phenolate, which are ubiquitous biologically relevant structural motifs whose vertical ionization and detachment energies (VIEs and VDEs) have been determined using X-ray LJ-PES.^42^ Our procedure takes only a few seconds to run on a standard laptop computer and will be straightforward to apply to time-resolved LJ-PES measurements comprised of tens of individual data sets.

Our retrieval method assumes that true photoelectron distributions can be represented by a weighted sum of Gaussian functions, *I*_true_(*E*) = ∑_*i*_*c*_*i*_*G*_*i*_(*E*), where each Gaussian *G*_*i*_(*E*), with weight *c*_*i*_, has its own central eKE and full width at half-maximum (FWHM). Following the approach of Suzuki and co-workers,^26^ we then assume that the measured UV photoelectron spectra can be fit to a linear combination of *g*_*i*_(*E*), given by *I*_meas_(*E*) = ∑_*i*_*c*_*i*_*g*_*i*_(*E*), where *g*_*i*_(*E*) are the measured eKE profiles representing the effect of distortion by inelastic scattering on the initial Gaussian distributions *G*_*i*_(*E*). In contrast to Suzuki and co-workers, who use an EUV PES measurement to determine *G*_*i*_(*E*) → *g*_*i*_(*E*) transformations, we derive the *G*_*i*_(*E*) → *g*_*i*_(*E*) transformations from Monte Carlo simulations of the electron transport equations in water, using an algorithm similar to those used by Signorell, Wörner, Green, and corresponding co-workers.^25,43,52^

The *G*_*i*_(*E*) → *g*_*i*_(*E*) transformations are built “on-the-fly” from a basis set of *E*_*z*_ → *S*_*z*_(*E*) transformations, where *E*_*z*_ is the initial eKE of an electron formed at a distance *z* from the liquid–vacuum interface and *S*_*z*_(*E*) is the eKE distribution that leaves the liquid. Example *E*_*z*_ → *S*_*z*_(*E*) transformation functions are presented in Supporting Information Figure S4. Currently, our model neglects anisotropy, but the transformation functions could provide angular information by including the polarization vector of the laser pulse and angular dependence of the scattering cross-sections. The liquid jet is assumed to be illuminated uniformly by UV light, on the basis of previously reported numerical simulations of UV light propagation through water.^25^ In contrast to the case of water, where nascent photoelectrons are assumed to be formed with uniform probability throughout the microjet, the depth profiles of solutes and their corresponding photoelectrons are taken into account by weighting the sampling of electron formation locations by the solute probability density as a function of depth beneath the surface of the microjet. Guided by the results of molecular dynamics trajectories of dilute phenol and phenolate aqueous solutions, summarized in the Supporting Information,^44,45^ we use an exponential function with a mean 0.5 nm below the water surface to describe the concentration profiles of phenol and phenolate in aqueous solution (Figure S6). Fitting the measured photoelectron kinetic energy distribution, *I*_meas_(*E*), to the linear combination ∑_*i*_*c*_*i*_*g*_*i*_(*E*) was performed using a Levenberg–Marquardt least-squares algorithm. The data were fit above 0.20 eV (water), 0.20–0.35 eV (phenol), or 0.15 eV (phenolate). The different cutoffs for different sets of data arise from both the correction for the transmission function of our photoelectron spectrometer (Figure S1) and our correction for the vacuum-level offset (Figure S2). The fitting procedure takes several seconds on a laptop computer, including the on-the-fly construction of *G*_*i*_(*E*) → *g*_*i*_(*E*) transformations. The *E*_*z*_ → *S*_*z*_(*E*) transformation functions take several minutes to an hour to compute and are saved to disc for reuse.

We demonstrate the wide applicability of our spectral retrieval method by determining accurate measurements of the lowest VIE of liquid water and the lowest VIE and VDE of phenol and phenolate in aqueous solution, respectively. A two-photon, non-resonant photoelectron spectrum of liquid water was recorded at 200.2 nm under near-zero streaming potential conditions, corrected for the instrument function of the photoelectron spectrometer and the vacuum-level offset between the aqueous solution and the detector, and plotted as a function of eKE (Figure 2a; see also Table 1). The measured spectrum has a profile that is almost Gaussian, with a slight skew toward lower eKE, a peak maximum of 0.83 ± 0.07 eV, and FWHM = 1.20 eV. Our spectral retrieval method was applied and the fitted spectrum is plotted over the experimental data, with the residuals plotted below, together with the retrieved photoelectron spectrum. The retrieved photoelectron spectrum has a peak maximum of 1.03 ± 0.07 eV and FWHM = 1.27 eV, corresponding to a vertical ionization energy, VIE = 2*hν* – eKE = 11.36 ± 0.09 eV. This value is in agreement with recently reported values obtained from X-ray photoelectron spectroscopy measurements (average of measurements made at a range of wavelengths, 11.33 ± 0.03 eV)^41^ and EUV photoelectron spectroscopy measurements made with a helium discharge lamp (11.40 ± 0.07 eV)^40^ (Figure 2b). The VIE determined from our raw data is overestimated by 0.2 eV as a result of inelastic scattering of low-energy electrons; however, it is worth noting that VIEs derived from experiments using EUV photon energies around 15 eV can be overestimated by as much as 0.5 eV due to electronically inelastic scattering.^41^ There has been some debate about the effect of the liquid–vacuum interface escape threshold, which is related to the electron affinity of water, on Monte Carlo simulations of liquid photoelectron spectra.^27,28^ Signorell showed that varying the escape threshold from 1.0 to 0.1 eV did not affect the maximum of the retrieved eBE distribution of the *e*_aq_^–^ photoelectron spectrum,^28,29^ and we have found that varying the escape threshold from 1.0 to 0.1 eV has little impact on the retrieved VIE of liquid water, reducing it by only 0.04 eV (Figure S5), which is still in good agreement with recent literature values.^40,41^

**Figure 2:**
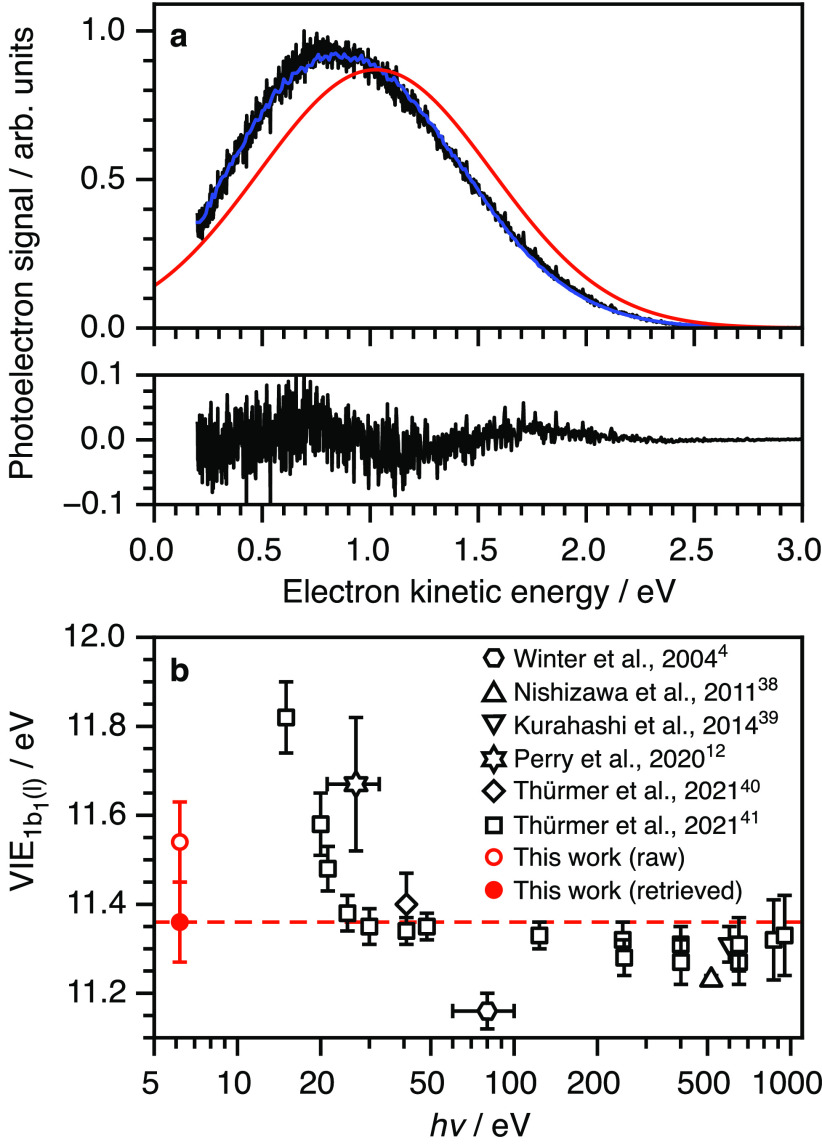
(a) Photoelectron spectrum of water following nonresonant two-photon ionization at 200.2 nm (black) together with the fit to *I*_meas_(*E*) (blue) and corresponding retrieved *I*_true_(*E*) distribution (red). The residuals associated with the fit are plotted below the spectrum, and the measured and retrieved electron kinetic energies and binding energy are presented in Table 1. (b) Plot of values of the 1b_1_ vertical ionization energy of liquid water as a function of photon energy. Data from refs (4, 12, and 38−41).

**Table 1:** Peak Maxima (eKE) of *I*_meas_(*E*) and *I*_true_(*E*), Full Widths at Half-Maxima (FWHMs) of eKE_true_, and One- and Two-Photon Binding Energies, *hν* – eKE and 2*hν* – eKE, Obtained from the Photoelectron Spectra of Water, Phenol, and Phenolate Presented in Figures 2 and 3^a^

molecule	λ/nm	eKE_meas_/eV	eKE_true_/eV	FWHM_true_/eV	*hν* – eKE/eV	2*hν* – eKE/eV
water	200.2	0.83 ± 0.07	1.03 ± 0.07	1.27	5.16 ± 0.07	**11.36** ± **0.09**
phenol	290.0	0.67 ± 0.07	0.79 ± 0.07	0.94		**7.76** ± **0.09**
	278.6	0.70 ± 0.07	0.73 ± 0.07	0.90	3.72 ± 0.07	8.17 ± 0.09
	272.5	0.73 ± 0.07	0.77 ± 0.07	0.92	3.78 ± 0.07	8.33 ± 0.09
	266.6	0.76 ± 0.07	0.81 ± 0.07	0.96	3.84 ± 0.07	8.49 ± 0.09
phenolate	320.0	0.62 ± 0.07	0.73 ± 0.07	0.87		**7.02** ± **0.09**
	298.7	0.92 ± 0.07	0.98 ± 0.07	0.98	3.14 ± 0.07	7.32 ± 0.09
	291.7	0.95 ± 0.07	1.01 ± 0.07	1.04	3.21 ± 0.07	7.49 ± 0.09
	285.0	1.00 ± 0.07	1.06 ± 0.07	1.09	3.29 ± 0.07	7.64 ± 0.09

a Vertical ionization and detachment energies arising from non-resonant excitation are in bold.

Multiphoton photoelectron spectra of aqueous phenol and phenolate were recorded following photoexcitation at a range of wavelengths just below the onset of, or resonant with, their S_0_ → S_1_ transitions and are plotted as a function of eKE in Figure 3). Because these weakly soluble organic molecules tend to have an enhanced concentration at the liquid–vacuum interface, we applied our spectral retrieval method using an exponential solute concentration depth profile. The spectra presented in Figure 3a,f are non-resonant multiphoton ionization/detachment spectra obtained by subtracting solvent-only spectra to isolate the organic chromophore contributions. The retrieved photoelectron spectra have peak maxima of 0.79 ± 0.07 and 0.73 ± 0.07 eV, corresponding to vertical ionization/detachment energies VIE/VDE = 2*hν* – eKE = 7.76 ± 0.09 and 7.02 ± 0.09 eV, which are in agreement with X-ray photoelectron spectroscopy measurements, 7.8 ± 0.1 and 7.1 ± 0.1 eV,^42^ respectively. It is worth noting that the values obtained using the peak maxima of the measured distributions, *I*_meas_(*E*), are only overestimated by around 0.1 eV. This is less than the overestimate observed in the UV photoelectron spectrum of water because the photoelectrons originate predominantly from within a nanometer of the surface of the liquid–jet and undergo very few scattering events before being detected. This observation is consistent with the results of our recent photoelectron spectroscopy study of the green fluorescent protein chromophore in aqueous solution, in which one-dimensional electron scattering simulations suggested that the eKE loss was <0.2 eV.^14^

**Figure 3:**
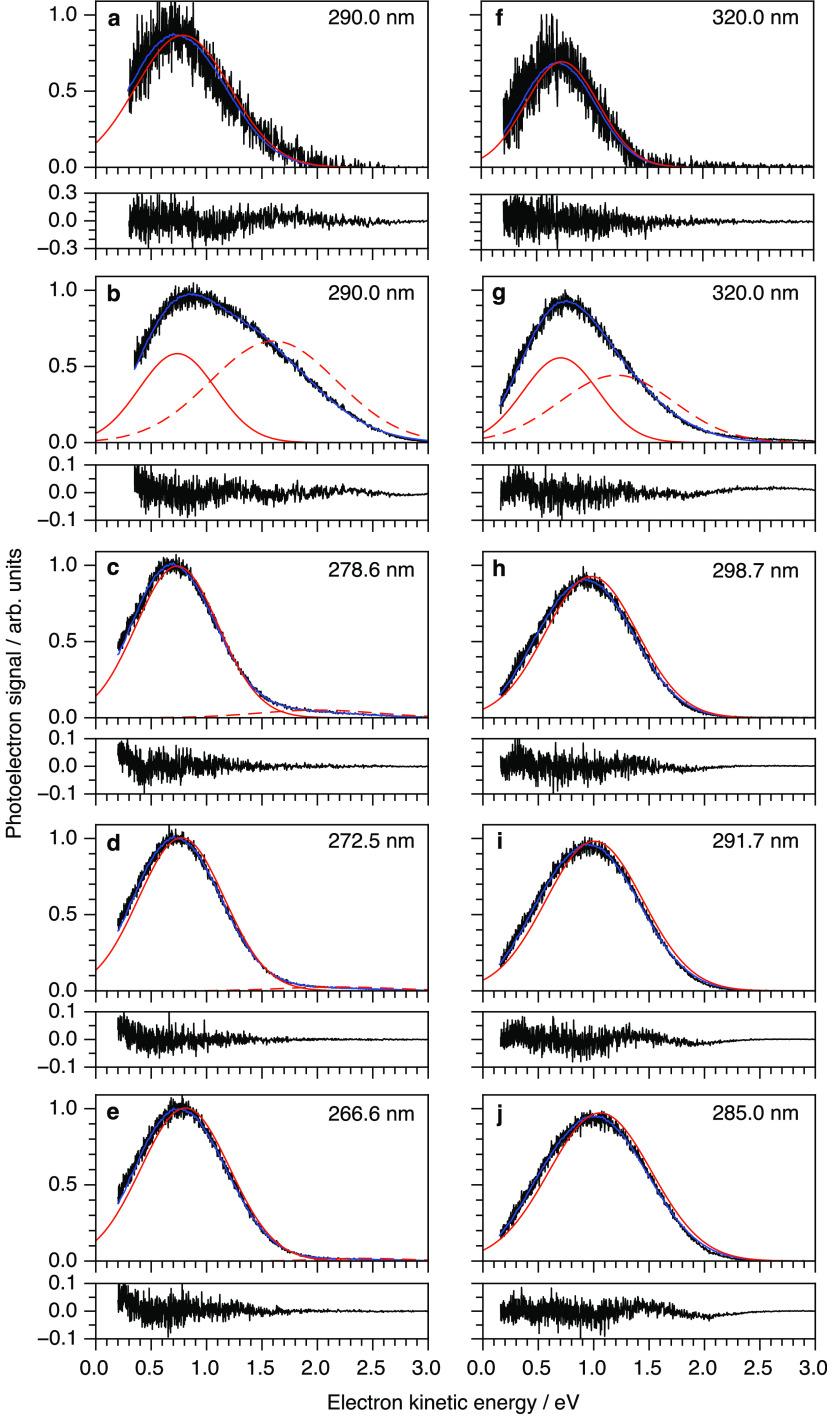
Photoelectron spectra of phenol (a–e) and phenolate (f–j) following multiphoton ionization/detachment (black) together with the fits to *I*_meas_(*E*) (blue) and corresponding retrieved *I*_true_(*E*) distributions (red). The residuals associated with the fits are plotted below the spectra. Photoelectron spectra presented in panels a and f are nonresonant multiphoton ionization/detachment spectra obtained by subtracting solvent-only spectra to isolate the organic chromophore contributions; the corresponding original spectra are presented in panels b and g. Photoelectron spectra presented in panels c–e and h–j are resonant multiphoton ionization/detachment spectra. There are weak contributions from three-photon ionization of liquid water to the spectra of aqueous phenol in panels c–e (dashed red lines).

Next, we consider the resonance-enhanced photoelectron spectra presented in Figure 3c–e,h–j. For both molecules, the retrieved two-photon binding energies rise monotonically as the photon energy is increased to scan over the S_1_ band, from 8.17 to 8.49 eV for phenol and from 7.32 to 7.64 eV for phenolate. All of the *I*_true_(*E*) profiles have FWHM of approximately 1 eV, consistent with previously reported X-ray LJ-PES spectra;^42^ however, they also increase slightly with increasing photon energy. We attribute both the increase in two-photon binding energy and the FWHM to vibrational relaxation within the S_1_ states or changing Franck–Condon profiles, since it is known that electronic relaxation of the S_1_ states is longer than the pulse duration of our laser pulses.^46,47^ Full analyses of these spectra, supported by accurate quantum chemistry calculations, are underway.

We now return to the non-resonant photoelectron spectra presented in Figures 3b,g. These spectra have contributions from both the solvent and solute and are best fit to two initial Gaussian distributions with different concentration depth profiles. The lower eKE features are attributed to phenol or phenolate (with exponential concentration depth profiles) and the higher eKE features are attributed to the solvent (with uniform concentration depth profiles). We note that only fitting with the sum of two features gives an adequate description of the peak shape (Figure S8).

For phenol (Figure 3b), the lower eKE feature has eKE_true_ = 0.74 ± 0.07 eV, which corresponds to a VIE of 7.81 ± 0.09 eV. This value is in good agreement with the VIE of 7.76 ± 0.09 eV extracted from the background-subtracted spectrum. The higher eKE feature has eKE_true_ = 1.61 ± 0.07 eV, which we attribute to three-photon ionization of water. The three-photon binding energy of the background signal in the 290.0 nm spectrum of aqueous phenol is 11.2 ± 0.1 eV. We suspect that the difference between the VIE determined from three-photon ionization and that obtained by two-photon nonresonant ionization at 200.2 nm (Figure 2) is a resonance in the absorption spectrum of water at the two-photon-level^43,48,49^ shifting the Franck–Condon profile. Interestingly, there are also weak contributions from bulk water signals observed in each of the three resonant spectra of phenol that have been fit using bulk solute distributions centered at 2.15 ± 0.07, 2.32 ± 0.07, and 2.50 ± 0.07 eV, corresponding to three-photon binding energies of 11.2 ± 0.1, 11.3 ± 0.1, and 11.5 ± 0.1 eV.

For phenolate (Figure 3g), the lower eKE feature has eKE_true_ = 0.70 ± 0.07 eV, which corresponds to a VIE of 7.05 ± 0.09 eV. Again, this value is within error of the VIE obtained from the background-subtracted spectrum, even with the complication of a second feature within the same LJ-PES spectrum. The higher eKE feature has eKE_true_ = 1.21 ± 0.07 eV, which corresponds to a three-photon binding energy of 10.41 ± 0.09 eV. Three-photon ionization of liquid water is not possible at this wavelength. The most plausible explanation is 2 + 1 resonance-enhanced detachment via a high-lying electronically excited state of aqueous hydroxide, which is present at a concentration of 2.0 mM in the aqueous solution of phenolate and has a VDE of 9.2 eV.^50^

In summary, we have developed an efficient and widely applicable method for retrieving true photoelectron spectra from UV LJ-PES measurements of liquids to an accuracy of ≲0.1 eV; this is comparable to state-of-the-art LJ-PES measurements employing X-ray or EUV light,^25,26,41^ which require synchrotrons or high-harmonic generation. As a result, UV LJ-PES is now ready to become a powerful technique for accurate determination of valence electronic structure of molecules in aqueous solution, which is especially important for sparingly soluble organic molecules for which X-ray LJ-PES is not feasible due to overwhelming contributions from solvent. This capability is not only significant in terms of enabling us to improve our fundamental understanding of the electronic structure and dynamics of aqueous solutions of organic molecules but also provides new opportunities for quantitative studies of low-energy (<5 eV) electron scattering events in fields such as radiation biology and chemistry, atmospheric science, nuclear energy, plasma medicine, and plasma water purification. The possibility of retrieving true photoelectron spectra of components of a solution with different concentration depth profiles suggests that UV LJ-PES also has the capability to become a useful analytical tool. Our analysis also shows that the low-energy scattering cross-sections (<3 eV) derived from photoelectron imaging of water nanodroplets,^31^ that have been subject to debate,^25,32,43,51^ are robust.

## Methods

Photoelectron spectra were recorded using our liquid-microjet magnetic-bottle time-of-flight (TOF) photoelectron spectrometer.^21^ Solutions were prepared using ultrapure water (Purelab Chorus, Elga, >15 MΩ·cm), with 0.8 mM NaF to minimize charging of the jet (liquid water measurement), 0.1 mM phenol and 1.75 mM of NaF (aqueous phenol measurements) or 0.1 mM phenol and 2.0 mM NaOH (aqueous phenolate measurements). These solutions were introduced into the source chamber of the photoelectron spectrometer through a 20 μm diameter fused silica capillary (AdMiSys) using a high-performance liquid chromatography pump (backing pressure, 65–75 bar; flow rate, 0.7 mL/min). Around 2 mm downstream from the capillary nozzle, the resulting laminar jet was intersected by femtosecond laser pulses generated by an optical parametric amplifier (Coherent OPerA Solo) pumped by a regenerative amplifier (Coherent Astrella-HE), before the liquid was collected in a warmed beryllium copper catcher and removed from the vacuum system using a peristaltic pump. Photoelectrons resulting from multiphoton ionization or detachment were guided into the TOF photoelectron spectrometer by a strong inhomogeneous magnetic field (∼1 T). The photoelectron spectrum was built up as a histogram of electron counts against TOF to a microchannel plate detector. Photoelectron spectra of NO and Xe were recorded to convert TOF to eKE and to determine the energy resolution (Δ*E*/*E* ∼ 1%), instrument function, streaming potential, and vacuum-level offset between the interaction region and analyzer, the procedures for which are described in detail in the Supporting Information.

## Supporting Information Available

Time-of-flight to kinetic energy conversion; instrument transmission function; streaming potential and vacuum-level offset; *E*_*z*_ → *S*_*z*_(*E*) transformation function; solute concentration depth profiles; spectral retrieval; uncertainty analysis; example application to solvated electron spectrum (PDF)
Transparent Peer Review report available (PDF)

## Notes

The software will be made available to the scientific community. Requests should be submitted to h.h.fielding@ucl.ac.uk.
