# Supplementary material for: Accurate Vertical Ionization Energy of Water and Retrieval of True Ultraviolet Photoelectron Spectra of Aqueous Solutions
Source: J Phys Chem Lett. 2022 Jul 21;13(30):6889–95. doi: 10.1021/acs.jpclett.2c01768 (PMC9358712; doi:10.1021/acs.jpclett.2c01768)
Supplement: Supplementary file 1 — jz2c01768_si_001.pdf [file jz2c01768_si_001.pdf]

Supplementary Information

Accurate Vertical Ionization Energy of Water and Retrieval of True Ultraviolet Photoelectron Spectra of Aqueous Solutions

Michael S. Scholz, William G. Fortune, Omri Tau, and Helen H. Fielding[1]

Department of Chemistry, University College London, 20 Gordon Street, London, WC1H 0AJ, U.K.

E-mail: h.h.fielding@ucl.ac.uk

[1]To whom correspondence should be addressed

S1 Time-of-flight to kinetic energy conversion

Time-of-flight (ToF) to electron kinetic energy (eKE) calibration was carried out by employing 2+1 REMPI of Xe at 249.7 nm and non-resonant MPI of NO over 237–266 nm to obtain a series of time-of-flight spectra containing distinct transitions with well-known eKEs.[1,2] The TOF-to-eKE conversion was performed by least-squares fitting to these data. This calibration was performed every time experimental conditions inside the vacuum chamber changed, for example, when the microjet nozzle graphite coating was reapplied.

S2 Instrument transmission function

The instrument transmission function was determined by recording non-resonant photoelectron spectra of NO at a number of wavelengths spanning 237–266 nm. The relative signal loss compared to the 0-0 vibronic band is plotted as a function of eKE to yield the data plotted in Figure S1. An exponential decay fit to these data points yields the instrument transmission function, which we use to correct our spectra.

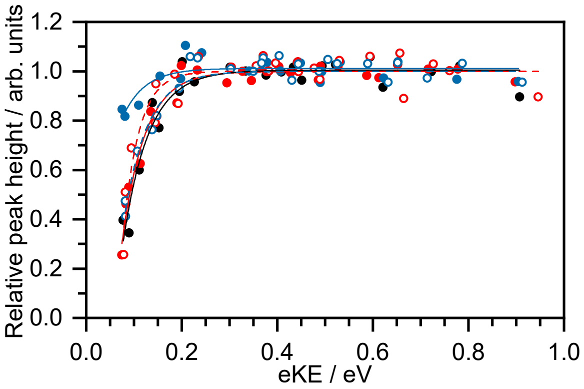

Figure S1: Relative peak heights for non-resonant two-photon photoelectron spectra of NO between total photon energies of 9.93 eV $\leq h\nu \leq$ 10.46 eV. Solid lines are exponential fits to each data set. The instrument function is sensitive to the quality of the graphite coating of the interaction region and we have found that it deteriorates with time. When it becomes less acceptable, we reapply the graphite coating. We have shown all the instrument functions determined for the data reported in this work: the black, red, and blue circles correspond to data recorded for water, phenol, and phenolate, respectively; the open circles and dashed lines indicate data and fits for treating the background-subtracted non-resonant spectra of phenol and phenolate. Photoelectron spectra were transformed with the relevant instrument transmission function following collection.

S3 Streaming potential and vacuum level offset

The streaming potential of the liquid jet is determined routinely at the start and end of each day and upon changing laser wavelength. The potential is determined by measuring the shift in electron kinetic energy of the Xe $^2P_{3/2}$ signal as a function of the distance between the microjet and the gas ionization point. The vacuum level offset is obtained from the asymptotic eKE value as the liquid jet is moved away from the gas ionization point through comparison with the field-free Xe $^2P_{3/2}$ value. Typical streaming potential measurements are presented in Figure S2.

The streaming potential was adjusted close to zero by addition of millimolar concentrations of an electrolyte, 2.0 mM NaOH or 1.75 mM NaF for phenolate and phenol, respectively. All spectra

measured on a single day were corrected using the average streaming potential and vacuum level offset of that day and the variance in these is taken into account in our error analysis.

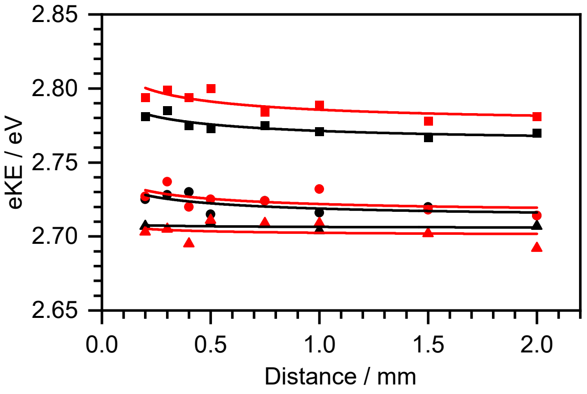

Figure S2: Experimentally determined streaming potentials recorded in the presence of phenolate (black) and phenol (red) as a function of the distance between the ionization point and the liquid microjet, recorded before (squares), during (circles) and after (triangles) collection of photoelectron spectra presented in Figure 3 of the paper. All streaming potentials displayed have magnitude < 0.1 eV.

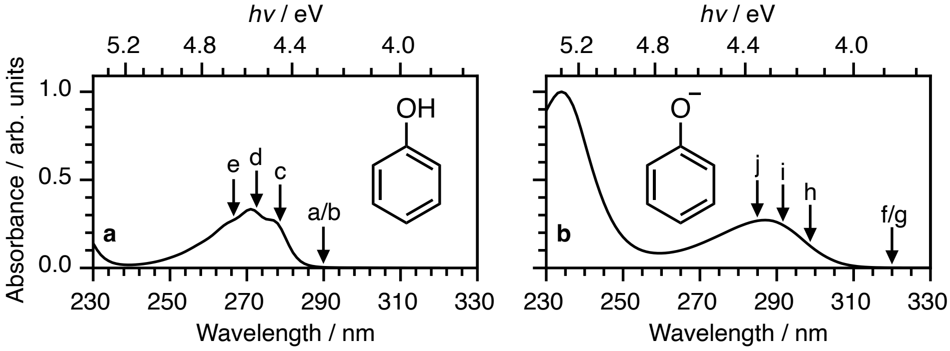

Figure S3: UV-visible absorption spectra of phenol and phenolate. Arrows mark the wavelengths used to record the LJ-PES reported in the main text.

S4 $E_z \rightarrow S_z(E)$ transformation functions

At the heart of our spectral retrieval software is a series of transformation functions which describe the probability distribution of yielding final eKEs, $S_z(E)$, for an electron with a given initial eKE and depth, E_z, following inelastic scattering by solvent. These transformations are generated by solving the transport equations for aqueous electrons employing a Monte Carlo procedure similar to those of Green, Signorell, Wörner, and respective coworkers.[3–5] Example $E_z \rightarrow S_z(E)$ transformation functions for exponential solute and uniform solvent distributions are presented in Figure S4.

Electrons are initialised within a water cylinder with probing depths sampled uniformly between the surface and the centre of the jet, typically in 0.1–5.0 nm steps, depending on the solute concentration profile. Their initial kinetic energy is given by E_z, which we vary over 0–5 eV in 0.01 eV steps. The initial direction and all following steps are sampled from uniform isotropic distributions. Although our model neglects the anisotropy of $I_{\text{meas}}(E)$, our $E_z \rightarrow S_z(E)$ transformation functions could be extended to provide angular information by including the angular dependence of the scattering cross-section. This could be implemented, for example,

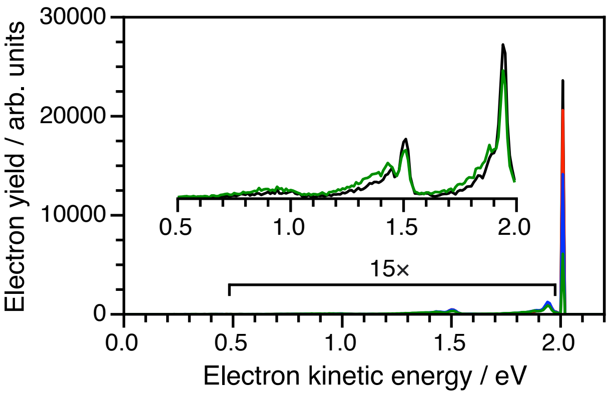

Figure S4: Example $E_z \rightarrow S_z(E)$ transformation functions for $E_z = 2.0$ eV. Electrons were initialised at $z = 1$ nm (black), 3 nm (red), 10 nm (blue), and 30 nm (green), with 50 000 electrons propagated at each depth. The inset depicts the 1 nm and 30 nm functions magnified by 15× for clarity.

by extending the transformation functions to have form $E_z(\theta) \rightarrow S_z(E, \theta)$, where θ is the laser polarization vector in the laboratory frame. Indeed, this is something we plan to do if we observe angular dependence in future measurements.

The initialised electrons undergo random walks, with step lengths chosen from an exponential distribution such that the mean value is governed by a randomly-selected elastic or inelastic scattering channel. These scattering channel probabilities are weighted by their relative cross-section at the current kinetic energy. We employ the water nanodroplet photoelectron spectroscopy data of Signorell and coworkers and the amorphous ice electron energy loss spectroscopy (EELS) data of Michaud and Sanche,[4,6] as digitised from Reference 4. If the step takes the electron out of the jet with positive kinetic energy, the trajectory is ended and the final eKE value saved. Otherwise, the electron loses energy, with the value sampled from a normal distribution based on the experimental phonon energies and linewidths from EELS spectra. Electrons that drop to zero kinetic energy are discarded and those with positive energies take another random step.

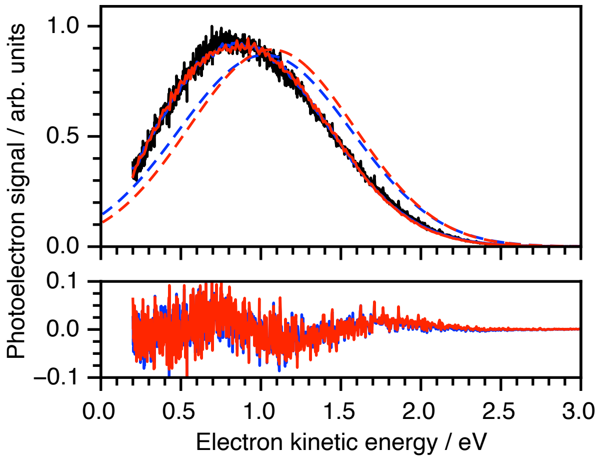

Figure S5: Effect of varying the electron affinity on the fit to the water photoelectron spectrum at 200.2 nm (black). The fit to the measured spectrum $I_{\text{meas}}(E)$ and retrieved $I_{\text{true}}(E)$ are plotted as solid and dashed lines, respectively. Changing E_{ea} from 1.0 eV (blue) to 0.1 eV (red) shifts the retrieved eKE_{true} by 0.04 eV to lower eKE.

The electrons that leave the jet with positive eKE are filtered using an escape threshold

function $T(E)$, which models the likelihood that an electron with a given kinetic energy can escape from the liquid jet into vacuum. We use the formulation of Michaud and Sanche:[6]

$$T(E) = 1 - \sqrt{\frac{E_{\mathrm{ea}}}{E + E_{\mathrm{ea}}}} \tag{1}$$

where E_{ea} is the electron affinity of water, which we set to 1.0 eV following the work of Signorell and coworkers.[4] The retrieval is only slightly sensitive to the value of E_{ea}; $\mathrm{eKE_{true}}$ is shifted only 0.04 eV to higher eKE when using $E_{\mathrm{ea}} = 0.1$ eV (Figure S5), a value suggested by Bartels.[7] This supports work by Signorell in which she showed that varying the escape threshold from 1.0 eV to 0.1 eV did not affect the maximum of the retrieved eBE distribution of the e^-_{aq} photoelectron spectrum.[8,9]

S5 Solute concentration depth profiles

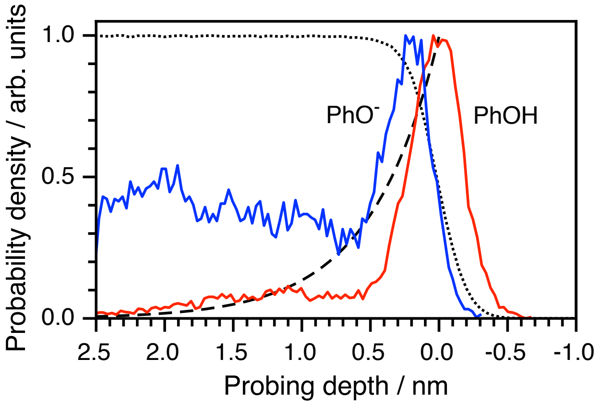

Figure S6: Probability densities obtained from molecular dynamics simulations of liquid-vacuum interfaces of water (black), and aqueous phenol (red) and phenolate (blue), as a function of the probing depth beneath the surface of the liquid jet. The water probability density is defined by the oxygen coordinates, whereas those of phenolate and phenol are based on the centre-of-mass coordinates. The exponential profile used to model the solute concentration is depicted by the dashed black line.

For the retrieved spectra of phenol and phenolate in the main text, we employ exponential distributions with mean 0.5 nm below the surface of the liquid-jet. This choice of profile was based on the results of molecular dynamics simulations of dilute solution-vacuum interfaces, with a single phenolate or phenol molecule per unit cell (Figure S6). The molecular dynamics simulations suggest that these small organic chromophores tend to be concentrated at the surface, even in the absence of π-stacking interactions. We note that at higher concentrations, phenol has a tendency to form dimers or trimers in solution, although only monomers have been detected near the vacuum-liquid interface.[10] The probability density distribution of phenol is dominated by a single peak within 1 nm of the liquid-vacuum interface. For phenolate, the probability density distribution peaks near the liquid-vacuum interface, although a significant fraction resides deeper within the solution (below 1 nm under the surface). These results are consistent with previous calculations for more concentrated phenol and phenolate solution-vacuum interfaces.[11,12] As a result, we expect that phenol displays behaviour somewhat closer to the limiting case where the molecules reside exclusively on the liquid surface, although phenolate should tend to also concentrate at the interface.

A comparison between exponential and Gaussian distributions with mean 0.5 nm below the liquid jet surface is presented in Figure S7. No difference between the spectra is discernible,

presumably because the mean free path of electrons is larger than the difference between the solute concentration functions. We note that non-ideal distributions such as those extracted from molecular dynamics simulations of water-vacuum interfaces could also be implemented.

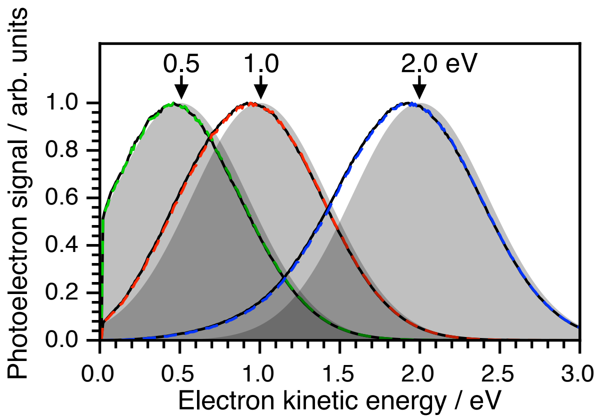

Figure S7: Comparison between exponential (dashed) and Gaussian (solid) solute distributions, both with means at 0.5 nm below the jet surface, superimposed over the input distributions with FWHM of 1 eV.

S6 $G_i(E) \rightarrow g_i(E)$ basis functions and $I_{\text{meas}}(E) \rightarrow I_{\text{true}}(E)$ retrieval

The $G_i(E) \rightarrow g_i(E)$ fitting basis functions are generated by taking the $E_z \rightarrow S_z(E)$ transformations, weighted by the concentration depth profile and normalised such that $\int I_{\text{meas}}(E) = 1$ for each E_z (typically E_z is 0–5.00 eV in 0.01 eV steps), and mapping them over a normal distribution with a given mean eKE and FWHM. The $I_{\text{meas}}(E) \rightarrow I_{\text{true}}(E)$ retrieval is then performed by fitting the experimental spectra to a linear combination of the $g_i(E)$ functions. We leave the amplitudes, positions, and optionally widths as free parameters in the fit, and can thereby retrieve $I_{\text{true}}(E)$. Currently, we employ the Levenberg-Marquardt least-squares non-linear fitting procedure implemented in LsqFit.jl.[13]

S7 Uncertainties in measurements and spectral retrieval

The accuracy of our UV LJ-PES measurements of organic chromophores is determined by a number of experimental factors: time-of-flight to kinetic energy conversion, instrument transmission function, streaming potential, and the vacuum level offset between the liquid-jet and the analyser. The retrieval of $I_{\text{true}}(E)$ also contains uncertainties, arising from the scattering cross sections, the electron affinity of water, the solute concentration profile, and the choice of function with which to represent $I_{\text{true}}(E)$ when fitting $I_{\text{meas}}(E)$.

We begin by discussing the experimental uncertainties. The central wavelength of our UV laser pulses is measured regularly using a spectrometer with absolute accuracy around ±0.02 eV. Using gas-phase photoelectron spectroscopy of Xe and NO to calibrate our time-of-flight spectrometer, we expect the uncertainty to be around ±0.03 eV. The instrument function of our electron spectrometer is measured routinely by comparing the relative heights of vibronic transitions in the photoelectron spectrum of gas-phase NO as a function of eKE. Transmission is observed to be unity for eKEs above 0.3 eV and greater than 80% above 0.15 eV (Figure S1). All data presented here are corrected for the transmission function, which was determined for each series of measurements, and as all peak maxima are well above 0.3 eV, we assume that the transmission function adds negligible uncertainty to our eKE_{meas} values. The streaming potential and vacuum level offsets were determined before, during and after every series of measurements

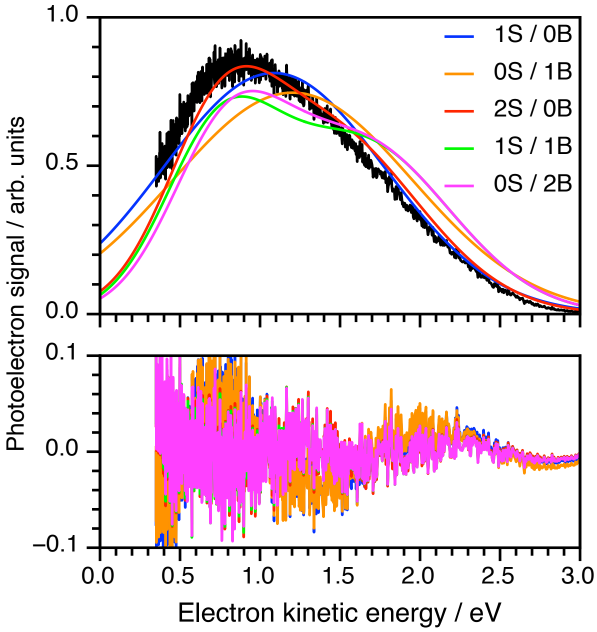

Figure S8: Comparison of fitting the 290.0 nm phenol LJ-PES spectrum with different numbers of bulk and surface contributions. MS / NB refers to the sum of M surface features and N bulk features. The retrieved I_{true} distributions are plotted above and the corresponding residuals below.

(Figure S2). The streaming potential is adjusted close to zero using salt (sodium hydroxide or sodium fluoride) and is measured to be around 0.05 ± 0.03 eV. The vacuum level offset between the liquid-jet and the analyser is observed to decrease consistently during the course of a series of measurements recorded in a single day, with an overall uncertainty of ± 0.05 eV (Figure S2). Overall, we estimate our propagated experimental uncertainty for $\mathrm{eKE_{meas}}$ to be around ± 0.07 eV for single-photon processes.

We now consider the uncertainties associated with the conversion of $I_{\mathrm{meas}}(E)$ to $I_{\mathrm{true}}(E)$. Signorell and coworkers have shown that the scattering cross-sections derived from photoelectron spectroscopy of water nanodroplets appear to be robust and are similar to those derived from spectroscopy of amorphous ice.[14] A change in retrieved $\mathrm{eKE_{true}}(E)$ of less than ± 0.01 eV is observed upon doubling or halving the scattering cross sections. Reducing the electron affinity of water in our model from 1.0 eV to 0.1 eV introduces a change in $\mathrm{eKE_{true}}$ of only ± 0.02 eV (Supplementary Text). We assume that the electron-water scattering resonance energies from amorphous ice are accurate to at least ± 0.01 eV.[6] The use of an exponential versus Gaussian solute concentration distribution introduces an uncertainty of around ± 0.01 eV for the surface-loving organic solutes studied here (Figure S5). Our choice of a Gaussian function to represent the $I_{\mathrm{true}}(E)$ profile is purely phenomenological, as we neglect vibronic structure; however, to the best of our knowledge, Gaussian functions have adequately described all LJ-PES spectra to date. Accumulated numerical errors arising from binning and rounding contribute ± 0.02 eV each. Overall, the errors arising from the conversion of $\mathrm{eKE_{meas}}(E)$ to $\mathrm{eKE_{true}}(E)$ are estimated to be around ± 0.04 eV.

S8 UV photoelectron spectrum of the solvated electron

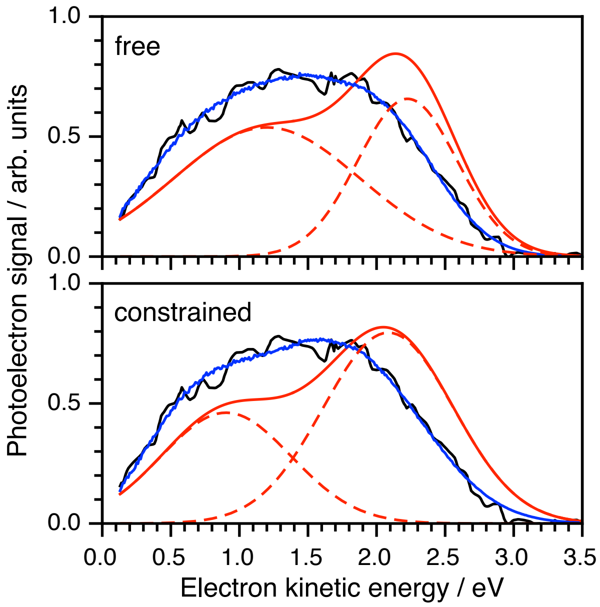

Figure S9: Example fits to solvated electron spectra that were recorded at $h\nu = 5.8$ eV and reported by by Signorell, Suzuki and coworkers.[4,15] The blue lines represent our fit and the red lines indicate $I_{\text{true}}(E)$. The spectra were best fit using the sum of two bulk Gaussian distributions, indicated by the dashed red lines, consistent with the previously reported retrieval from UV photoelectron spectra.[4] The top fit has both Gaussian widths free in the fit, where we constrain both widths to be identical in the bottom fit. We obtain eKE_{true} values of 3.7 ± 0.1 eV and 3.8 ± 0.1 eV for the free and constrained fits respectively, consistent with $I_{\text{true}}(E)$ retrieval from UV spectra (3.7 eV)[4] and accurate EUV measurements (3.76/3.77 eV).[16]

References

[1] Yoshino, K.; Freeman, D. *J. Opt. Soc. Am. B* **1985**, *2*, 1268–1274.

[2] Jarvis, G.; Evans, M.; Ng, C.; Mitsuke, K. *J. Chem. Phys.* **1999**, *111*, 3058–3069.

[3] Smith, M. E.; Green, N.; Pimblott, S. *J. Comput. Chem.* **2018**, *39*, 2217–2225.

[4] Luckhaus, D.; Yamamoto, Y.-I.; Suzuki, T.; Signorell, R. *Sci. Adv.* **2017**, *3*, e1603224.

[5] Schild, A.; Peper, M.; Perry, C.; Rattenbacher, D.; Wörner, H. J. *J. Phys. Chem. Lett.* **2020**, *11*, 1128–1134.

[6] Michaud, M.; Wen, A.; Sanche, L. *Rad. Res.* **2003**, *159*, 3–22.

[7] Bartels, D. M. *J. Phys. Chem. Lett.* **2019**, *10*, 4910–4913.

[8] Signorell, R. *J. Phys. Chem. Lett.* **2020**, *11*, 1516–1519.

[9] Signorell, R. *J. Phys. Chem. A* **2020**, *124*, 1666–1667.

[10] Kusaka, R.; Ishiyama, T.; Nihonyanagi, S.; Morita, A.; Tahara, T. *Phys. Chem. Chem. Phys.* **2018**, *20*, 3002–3009.

[11] Minofar, B.; Jungwirth, P.; Das, M. R.; Kunz, W.; Mahiuddin, S. *J. Phys. Chem. C* **2007**, *111*, 8242–8247.

[12] Sokhan, V.; Tildesley, D. *Faraday Discuss.* **1996**, *104*, 193–208.

[13] `LsqFit.jl`. `https://github.com/JuliaNLSolvers/LsqFit.jl`, Accessed: 2022-03-28.

[14] Signorell, R. *Phys. Rev. Lett.* **2020**, *124*, 205501.

[15] Yamamoto, Y.-I.; Karashima, S.; Adachi, S.; Suzuki, T. *J. Phys. Chem. A* **2016**, *120*, 1153–1159.

[16] Nishitani, J.; Yamamoto, Y.-i.; West, C. W.; Karashima, S.; Suzuki, T. *Sci. Adv.* **2019**, *5*, eaaw6896.
