# Supplementary material for: Accurate Vertical Ionization Energy of Water and Retrieval of True Ultraviolet Photoelectron Spectra of Aqueous Solutions
Source: J Phys Chem Lett. 2022 Jul 21;13(30):6889–95. doi: 10.1021/acs.jpclett.2c01768 (PMC9358712; doi:10.1021/acs.jpclett.2c01768)
Supplement: Supplementary file 2 — jz2c01768_si_002.pdf [file jz2c01768_si_002.pdf]

jz-2022-01768w.R1

Name: Peer Review Information for "Accurate Vertical Ionization Energy of Water and Retrieval of True Ultraviolet Photoelectron Spectra of Aqueous Solutions"

First Round of Reviewer Comments

Reviewer: 1

Comments to the Author

The manuscript by Scholz et al. reports on the determination of accurate ionization and detachment energies from liquid phase photoelectron spectra of neat water and water soluble organic substances (here phenol and phenolate). The main achievements of this work are: 1. the use of UV multiphoton ionization which provides an excellent signal to noise ratio, 2. the meticulous experimental work, in particular regarding calibration measurements etc., 3. the careful analysis of the experimental data explicitly accounting for the effects of inelastic electron scattering and solute concentration profiles. A particular highlight is the determination of the lowest ionization energy of neat liquid water (Fig. 2) at low photon energies. It finally confirms that the same value is found at low photon energies after correction for scattering as previously measured at high photon energies. This shows that recent claims to the contrary were wrong and caused by erroneous calibration and analysis.

The manuscript is very well written, the results are clear and well founded and of great scientific relevance. This is an excellent manuscript that clearly deserves publication in JPC Letters.

Before publication, I suggest that the authors take the following comments into consideration:

1. I would like to point the authors to the following recently published Perspective Article In PCCP, which covers the same subject; i. e. electron scattering and determination of accurate ionization energies in liquid phase photoelectron spectroscopy: doi: 10.1039/D2CP00164K. This references should be included in the introduction (line 49, left column).

2. The discussion of the previous approaches in the introduction (second column on p.1, line 16) might be misleading. The crucial advantage of the approaches of ref. 23 and 49 (should be cited here and at the end of the first paragraph on p.2 in line 41 left column) is not the use of the Monte Carlo technique, but the use of independent electron scattering cross sections that were previously determined from experimental droplet photoelectron spectra (see refs. 23, 49). This is important for the reader to understand and should therefore be made clearer in the introduction. This is the essential difference to the retrieval method of ref. 24,29,30 ,which includes no independent scattering information.

3. The approach proposed in this manuscript to account for the influence of electron scattering is definitely useful. In particular, the use of a library of Monte Carlo simulations for the linear decomposition of measured spectra is an elegant idea. However, it should be noted that the gain in computational speed of this approach compared with that of ref. 23 and 49 derives to a large extent

also from certain simplifications of the scattering model, i. e. the complete neglect of any anisotropies both in the nascent electron distribution and of the subsequent scattering events. It is plausible that these effects would not significantly change any of the results presented in this manuscript.

4. I suggest that the authors reconsider citing ref. 28 (line31, first column, p.2 and SI p.2). This contribution does not deal with sub-excitation electrons (< 7eV electron kinetic energy) moreover has major scientific flaws as discuss in arXiv:2001.06069.

5. Line 45, second columns, p.2, last sentence of the paragraph: It should be noted here that ref. 26 and 27 already demonstrated that a change in the escape barrier from 1.0 to 0.1 eV has no significant influence on the retrieved VIE. This should also be made clear in the SI at the top of p.4. The formulation there is misleading because Bartels in fact expected a major effect on the retrieved VIE which was proved wrong in refs. 26 and 27.

6. Lines 23-25, second column, p. 3: Perhaps it could be stated more explicitly that the photoelectrons originating from near the surface undergo only very few if any scattering events before being detected, and that therefore the VIE of surface enriched solutes (as for phenol and phenolate) is not much effected by scattering. For phenol and phenolate photoelectrons from within less than one nanometer and not “within a few nanometers” according to the assumed concentration profile (see SI Fig. 6). This does not hold for more homogeneously distributed solutes.

7. Line 18, first columns, p. 5: The interpretation of the 1.21 eKE_true feature seems not so obvious. As far as I can see the CTTS state itself could only be populated via 1 or 2-photon excitation. Does this mean that you propose a non-resonant 1+1 or 2+1 process overall? The signal seems surprisingly strong for that.

8. SI, p. 2, second paragraph of S4: Note that in ref. 6, the relevant phonon scattering is anisotropic (see Table 2, p.11 of ref. 6) so that it is not true that “most inelastic electron-phonon collisions obey s-wave behavior”. The statement “forward-scattering anisotropic collision will not affect the eKE distribution” is incorrect. This only applies to forward elastic scattering, but not inelastic scattering events.

Reviewer: 2

Comments to the Author

The manuscript of Fielding and coworkers clearly demonstrates a method firmly grounded in first principles physics to retrieve a true UV-PES spectrum from aqueous solutions. This study is very nicely carried out and excellently described; it develops a spectral retrieval method, using Monte Carlo simulation of electron scattering and the molecular dynamics to present a unifying method to alleviate the effect of inelastic scattering of electrons from the solvent molecules. The loss in eKE of the ejected electrons due to inelastic scattering, particularly for UV laser ionization schemes used in several labs around the world, often leads to spectral shift and distortions, making spectral analysis quite difficult.

While the current work used the similar method implemented by Suzuki and coworkers to transform the measured eKE spectrum to true eKE spectrum, it is better in that, following an approach of Signorell, it uses energy-dependent electron scattering cross sections to extract the photoelectron distributions instead of depending on a measured EUV or X-ray PES spectrum. Of particular note, the method is

described well enough for other researchers to reproduce, the approach includes the solute depth profile, and the paper provides enough examples to fully explore multiple issues at play, dealing head on with the signal arising from solvent. Critical analysis of results found from the spectral retrieval is included providing a robust and very encouraging conclusion that only fairly modest correction is actually needed to correct spectra. Significantly, the authors provide open access source code shared with the community, something absent from the contributions of Signorell and Suzuki. Overall, the paper is important and adds significant value to the growing field of liquid phase photoelectron spectroscopy and should be published in JPCL.

I would recommend that before publication, the authors consider the following minor points:

1. The authors mention "The data were fit above 0.20 eV (water), 0.20–0.35 eV (phenol) or 0.15 eV (phenolate), where we have confidence in our correction for the transmission function of our photoelectron spectrometer."

- The transmission function of the spectrometer is an instrument property, so why do the cutoff threshold spectra vary with time? As intensity data are corrected by the derived cut-off function, a brief analysis of the variation would be helpful.

2. The presented spectral retrieval method does not deal with anisotropy of the emitted photoelectrons, but authors suggested in SI that their method can extended to address anisotropic scattering and emission. It would be helpful if the authors could add couple of sentences in the main text to address this important point and to describe what modifications/extensions would be needed in their method/model to cover anisotropy as well.

3. The authors claim that the extra higher binding energy peak in the phenolate experiments comes from ionizing hydroxide through resonance with OH- CTTS band. I find this assignment suspect: the CTTS transition for hydroxide lies as substantially shorter wavelength (6.62 eV; Crowell, JCP 2004; band extends from 187 nm to 200 nm) than the photon wavelength used (320 nm). Moreover, the binding energy for aqueous hydroxide is very definitely not 2.6 eV, but closer to 9.2 eV (Winter, JACS 2006; Seidel, Ann. Rev. Phys. Chem. 2016). The citation for this CTTS band position given by the authors is not authorative; assignment from VUV work is to be trusted. This should be corrected and alternative assignment sort.

4. As a major motivation for the paper is the ability of UV LJ-PES to capture PE spectra of dilute organic chromophores, with respect to resonance-enhanced LJ-PES, there have been other reports in the literature as well as the authors' own work in 2018. It would be appropriate to cite Roy, et al., J. Phys. Chem. B 2018, and Kumar, Farad. Disc. 2018.

Author's Response to Peer Review Comments:

Manuscript ID jz-2022-01768w

We are delighted that the reviewers recognise the significance of our work, and are grateful for their careful reading of our manuscript and suggestions for improvements. Their suggestions are listed below (black) together with our responses (blue).

Reviewer 1

1. I would like to point the authors to the following recently published Perspective Article In PCCP, which covers the same subject; i.e. electron scattering and determination of accurate ionization energies in liquid phase photoelectron spectroscopy: doi: 10.1039/D2CP00164K. This references should be included in the introduction (line 49, left column).

   We have added this reference (Ref. 30 in revised manuscript).

2. The discussion of the previous approaches in the introduction (second column on p.1, line 16) might be misleading. The crucial advantage of the approaches of ref. 23 and 49 (should be cited here and at the end of the first paragraph on p.2 in line 41 left column) is not the use of the Monte Carlo technique, but the use of independent electron scattering cross sections that were previously determined from experimental droplet photoelectron spectra (see refs. 23, 49). This is important for the reader to understand and should therefore be made clearer in the introduction. This is the essential difference to the retrieval method of ref. 24,29,30 ,which includes no independent scattering information.

   We have edited the text in the second paragraph on p1 to stress that the Monte Carlo simulations employed previously determined electron scattering cross-sections and have referenced the droplet work at this point in the manuscript as well: "Monte Carlo simulations using electron scattering cross-sections determined from photoelectron spectroscopy measurements of liquid droplets have been employed ... ."

3. The approach proposed in this manuscript to account for the influence of electron scattering is definitely useful. In particular, the use of a library of Monte Carlo simulations for the linear decomposition of measured spectra is an elegant idea. However, it should be noted that the gain in computational speed of this approach compared with that of ref. 23 and 49 derives to a large extent also from certain simplifications of the scattering model, i.e. the complete neglect of any anisotropies both in the nascent electron distribution and of the subsequent scattering events. It is plausible that these effects would not significantly change any of the results presented in this manuscript.

   A large factor in the computational speed of our approach is the library of MC simulations. Since we did not measure the anisotropy, we did not feel it necessary to include it in our model. Nonetheless, it is something we are considering for a future version of our software, which we address below–response (8).

4. I suggest that the authors reconsider citing ref. 28 (line31, first column, p.2 and SI p.2). This contribution does not deal with sub-excitation electrons ($<$ 7eV electron kinetic energy) moreover has major scientific flaws as discuss in arXiv:2001.06069.

   We have replaced this reference with a more recent one from the same group that, to the best of our knowledge, appears to have addressed these flaws.

5. Line 45, second columns, p.2, last sentence of the paragraph: It should be noted here that ref. 26 and 27 already demonstrated that a change in the escape barrier from 1.0 to 0.1 eV has no significant influence on the retrieved VIE. This should also be made clear in the SI at the top of p.4. The formulation there is misleading because Bartels in fact expected a major effect on the retrieved VIE which was proved wrong in refs. 26 and 27.

   We have modified the text in the manuscript to clarify this, "Signorell showed that varying the escape threshold from 1.0 eV to 0.1 eV did not affect the maximum of the retrieved eBE

distributiion of the e^-_{aq} photoelectron spectrum, and we have found that varying the escape threshold from 1.0 eV to 0.1 eV has little impact on the retrieved VIE of liquid water, reducing it by only 0.04 eV (Figure S5) which is still in good agreement with recent literature values."

We also modified the text in the SI by adding an additional sentence, "This supports work by Signorell in which she showed that varying the escape threshold from 1.0 eV to 0.1 eV did not affect the maximum of the retrieved eBE distributiion of the e^-_{aq} photoelectron spectrum."

6. Lines 23-25, second column, p. 3: Perhaps it could be stated more explicitly that the photoelectrons originating from near the surface undergo only very few if any scattering events before being detected, and that therefore the VIE of surface enriched solutes (as for phenol and phenolate) is not much effected by scattering. For phenol and phenolate photoelectrons from within less than one nanometer and not "within a few nanometers" according to the assumed concentration profile (see SI Fig. 6). This does not hold for more homogeneously distributed solutes.

   We have edited the text to read, "... originate predominantly from within a nanometre of the surface of the liquid-jet and undergo very few scattering events before being detected. "

7. Line 18, first columns, p. 5: The interpretation of the 1.21 eKE_{true} feature seems not so obvious. As far as I can see the CTTS state itself could only be populated via 1 or 2-photon excitation. Does this mean that you propose a non-resonant 1+1 or 2+1 process overall? The signal seems surprisingly strong for that.

   We were suggesting a 2+1 resonance-enhanced process, but in light of comment (3) by Reviewer 2, agree that it is unlikely to be via the CTTS state but must be via a higher-lying electronic state. The revised text reads, "The higher eKE feature has $eKE_{true} = 1.21 \pm 0.07$ eV, which corresponds to a three-photon binding energy of 10.41 ± 0.09 eV. Three-photon ionisation of liquid water is not possible at this wavelength. The most plausible explanation is $2 + 1$ resonance-enhanced detachment via a high-lying electronically excited state of aqueous hydroxide, which is present at a concentration of 2.0 mM in the aqueous solution of phenolate and has a VDE of 9.2 eV. "

8. SI, p. 2, second paragraph of S4: Note that in ref. 6, the relevant phonon scattering is anisotropic (see Table 2, p.11 of ref. 6) so that it is not true that "most inelastic electron-phonon collisions obey s-wave behavior". The statement "forward-scattering anisotropic collision will not affect the eKE distribution" is incorrect. This only applies to forward elastic scattering, but not inelastic scattering events.

   We agree that we made a mistake in writing this and have removed this statement.

Reviewer 2

1. The authors mention "The data were fit above 0.20 eV (water), 0.20–0.35 eV (phenol) or 0.15 eV (phenolate), where we have confidence in our correction for the transmission function of our photoelectron spectrometer." - The transmission function of the spectrometer is an instrument property, so why do the cutoff threshold spectra vary with time? As intensity data are corrected by the derived cut-off function, a brief analysis of the variation would be helpful.

   The differences between the sets of data is not just the instrument function, but also the different vacuum level offsets, which varies as a function of time during a measurement, as we explained in the SI. We have rephrased the text in the manuscript at the bottom of p2, column 1 to make this clearer, "The different cutoffs for different sets of data arise from both the correction for the transmission function of our photoelectron spectrometer (Fig. S1) and our correction for the vacuum level offset (Fig. S2)."

   In addition, the instrument function is sensitive to the quality of the graphite coating of the interaction region and we have found that it deteriorates with time. When it becomes less acceptable, we reapply the graphite coating. We have added this explanation to the caption of

Fig. S1. Please note that we have also modified Fig. S1 so that it now shows all the instrument functions determined for the data reported in this work.

2. The presented spectral retrieval method does not deal with anisotropy of the emitted photoelectrons, but authors suggested in SI that their method can extended to address anisotropic scattering and emission. It would be helpful if the authors could add couple of sentences in the main text to address this important point and to describe what modifications/extensions would be needed in their method/model to cover anisotropy as well.

   We have added the following text to the SI, "Although our model neglects the anisotropy of $I_{\text{meas}}(E)$, our $E_z \rightarrow S_z(E)$ transformation functions could be extended to provide angular information by including the angular dependence of the scattering cross-sections. Indeed, this is something we plan to do if we observe angular dependence in future measurements. This could be implemented, for example, by extending the transformation functions to have form $E_z(\theta) \rightarrow S_z(E, \theta)$, where θ is the laser polarisation vector in the laboratory frame. In the manuscript, we have added a sentence to paragraph 3 in column 1 of p2, "Currently, our model neglects anisotropy, but the transformation functions could provide angular information by including the polarisation vector of the laser pulse and the angular dependence of the scattering cross-sections."

3. The authors claim that the extra higher binding energy peak in the phenolate experiments comes from ionizing hydroxide through resonance with OH- CTTS band. I find this assignment suspect: the CTTS transition for hydroxide lies as substantially shorter wavelength (6.62 eV; Crowell, JCP 2004; band extends from 187 nm to 200 nm) than the photon wavelength used (320 nm). Moreover, the binding energy for aqueous hydroxide is very definitely not 2.6 eV, but closer to 9.2 eV (Winter, JACS 2006; Seidel, Ann. Rev. Phys. Chem. 2016). The citation for this CTTS band position given by the authors is not authorative; assignment from VUV work is to be trusted. This should be corrected and alternative assignment sort.

   We have addressed this in our response to Reviewer 1 (7). We also changed the reference (Ref. 50 in revised manuscript) to the JACS 2006 measurement recommended by this reviewer.

4. As a major motivation for the paper is the ability of UV LJ-PES to capture PE spectra of dilute organic chromophores, with respect to resonance-enhanced LJ-PES, there have been other reports in the literature as well as the authors' own work in 2018. It would be appropriate to cite Roy, et al., J. Phys. Chem. B 2018, and Kumar, Farad. Disc. 2018.

   We apologise: several of the references that were supposed to have been listed in the first paragraph of the manuscript were missing and several were not relevant. We have corrected this and our list includes those mentioned above (Refs 9, 14-23 in revised manuscript). We would also like to point out that a couple of the references at the end of the 3rd paragraph were also not relevant, so we have removed those as well.

Additional change to text in the manuscript

Please note that we have corrected the text in the second paragraph of p3 to refer to the increase in two-photon binding energy, rather than the increase in eKE. We changed,

"Next, we consider the resonance-enhanced photoelectron spectra presented in Figs 3(c-e) and (h-j). For both molecules, the retrieved eKE_{true} values rise monotonically as the photon energy is increased to scan over the S_1 band, from 0.73 eV to 0.81 eV for phenol and 0.98 eV to 1.06 eV for phenolate. All the $I_{\text{true}}(E)$ profiles have FWHM of approximately 1 eV, consistent with previously-reported X-ray LJ-PES spectra; however, they also increase slightly with increasing photon energy. We attribute both the increase in eKE and the FWHM to vibrational relaxation within the S_1 states or changing Franck-Condon profiles, since it is known that electronic relaxation of the S_1 states is longer than the pulse duration of our laser pulses. Full analyses of these spectra, supported by accurate quantum chemistry calculations, are underway."

to,

"Next, we consider the resonance-enhanced photoelectron spectra presented in Figs 3(c-e) and (h-j). For both molecules, the retrieved two-photon binding energies rise monotonically as the photon energy is increased to scan over the S_1 band, from 8.17 eV to 8.49 eV for phenol and 7.32 eV to 7.64 eV for phenolate. All the $I_{\text{true}}(E)$ profiles have FWHM of approximately 1 eV, consistent with previously-reported X-ray LJ-PES spectra; however, they also increase slightly with increasing photon energy. We attribute both the increase in two-photon binding energy and the FWHM to vibrational relaxation within the S_1 states or changing Franck-Condon profiles, since it is known that electronic relaxation of the S_1 states is longer than the pulse duration of our laser pulses. Full analyses of these spectra, supported by accurate quantum chemistry calculations, are underway."
